# The effect of feeding black soldier fly larvae on growth performance, protein, and fat content of red hybrid tilapia (*Oreochromis* spp.)

**DOI:** 10.14202/vetworld.2022.2453-2457

**Published:** 2022-10-22

**Authors:** H. N. Aisyah, Z. A. R. Athirah, W. R. Hanani, S. S. Arshad, H. A. Hassim, M. F. Nazarudin, M. Y. Ina-Salwany

**Affiliations:** 1 Department of Veterinary Preclinical Sciences, Faculty of Veterinary Medicine, Universiti Putra Malaysia, 43400 UPM Serdang, Malaysia; 2 Laboratory of Sustainable Animal Production and Biodiversity, Institute of Tropical Agriculture and Food Security, Universiti Putra Malaysia, 43400 UPM Serdang, Selangor, Malaysia; 3 Aquatic Animal Health and Therapeutics Laboratory, Institute of Bioscience, Universiti Putra Malaysia, 43400 UPM Serdang, Selangor, Malaysia

**Keywords:** black soldier fly larvae, growth performance, *Oreochromis* spp, red hybrid tilapia

## Abstract

**Background and Aim:**

In the aquaculture industry, the crucial goal is to minimize production costs, especially feeding costs, without significant side effects. Black soldier fly larva (BSFL) is a locally available, eco-friendly, and sustainable source that is high in crude protein (42% dry matter [DM]) and fat (35% DM). This study aimed to determine the growth performance along with the composition of crude fat and protein in red hybrid fingerlings after the addition of BSFL into the diet.

**Materials and Methods:**

A total of 120 fingerlings of uniform size (mean initial weight of 1.46 ± 0.06 g) were randomly assigned to one of four groups (n = 10) (A, B, C, and D) per tank (1 m × 2 m × 1 m). For 21 days, Group A (control group) was fed with 100% commercial diet; Group B was fed with 90% commercial fish diet + 10% BSFL; Group C was fed with 80% commercial fish diet + 20% BSFL; and Group D was fed with 70% commercial fish diet + 30% BSFL. Feed efficiency, growth performance, and proximate composition analysis were performed on the fish.

**Results:**

The results displayed that the group with the highest BSFL percentage had a greater effect on protein and fat composition than the control group. The proximate composition analysis of fish-fed diet revealed that an increase in the level of BSFL inclusion increases the protein content in the fish. In comparison to the other groups, the experimental diet with 30% BSFL inclusion has the highest levels of crude protein (80.30% DM) and fat (2.90% DM).

**Conclusion:**

It is concluded that incorporating BSFL into a commercial diet for red hybrid tilapia fingerlings increased crude protein and fat composition, providing an alternative protein and fat source in fish diets.

## Introduction

The Malaysian ecosystem provides an abundance of resources for aquatic farming; therefore, aquatic products are prevalent in the diets of the Malaysian population. Fish products meet a large percentage of the needs of the world population and account for 15% of all animal protein consumed globally [1]. The per capita consumption of fish in the Southeast Asian region is approximately 3 times that of the global average. Moreover, the global consumption index of fish increased from 53.1 kg in 2011 to 61.1 kg by 2020 and is expected to rise in the future due to the alarming rise in the population [1], positioning Malaysia as one of the world’s top fish consumers. Furthermore, to meet high market demand, it is critical to produce fish with a consistent growth rate in addition to higher nutritional value.

The primary goal in the aquaculture industry is to reduce production costs, especially feeding expenses, while avoiding significant side effects. Commercial aquaculture relies heavily on a commercial fish diet, which is a crucial component and formulated for the optimum growth of particular fish species. Commercial fish feed usually includes fish meal and oil, corn grain, and soybean meal to provide adequate amino acids and fatty acid profiles [2]. However, the usefulness of these ingredients is not limited to livestock but also humans. Therefore, due to the competition with humans, consumption, and limited stock, the price of these ingredients rises, increasing feeding and production costs. As a result, researchers and farmers are seeking alternatives to fish meal and oil, corn grain, and soybean meal, such as insect meal [3, 4]. There is a surge of interest in using it in animal feed as consumers become more aware of its advantages, including being a good source of protein and fat, consuming decaying organic matter, having a lower feed conversion ratio (FCR), and emitting fewer greenhouse gases [5]. Furthermore, these products will contribute to the reduction of the reliance on fish meal and oil, corn grain, and soybean meal.

The black soldier fly larva (BSFL) (*Hermetia illucens*) provides high crude protein (42%) and fat (35%) content [6, 7]. Studies have reported the usage of BSFL in animal diets such as swine, poultry, and the aquaculture industry [8-13]. In finishing pigs, BSFL may be included up to 4% of the diet [8], while in Japanese quails (*Coturnix coturnix japonica)*, BSFL may replace fish meal up to 50% [9]. In aquaculture, BSFL can replace fish meal in the diets of Pacific white shrimp (*Litopenaeus vannamei*) up to 25% [10], yellow catfish (*Pelteobagrus fulvidraco)* up to 48% [11], and juvenile barramundi (*Lates calcarifer*) and Nile tilapia (*Oreochromis niloticus*) up to 50% [12, 13]. Soy and corn protein sources are being researched for use in aquafeeds. However, research on the use of BSFL as a partial replacement for corn grain and soybean meal in aquatic feed is limited.

This study aimed to explore the possibility of using BSFL as a protein and fat resource in feeding red hybrid tilapia (as a partial replacer of corn grain and soybean meal) and to determine its effect on growth performance and protein and fat composition in red hybrid tilapia.

## Materials and Methods

### Ethical approval

All animals used in the present experiment were treated humanely, according to approved procedures and guidelines of the Universiti Putra Malaysia Animal Ethics Committee guidelines. The number of animals used in the present experimentation was approved by the committee.

### Study period and location

The study was conducted from 29-08-2020 to 19-09-2020. The red hybrid tilapias were purchased from a private fish hatchery in Balakong, Selangor, Malaysia, and were transported in continuously aerated polyethylene bags to the Aquatic Animal Health and Therapeutics Laboratory, Institute of Bioscience, Universiti Putra Malaysia (UPM), Serdang research facility. For two weeks, fish were acclimated to the experimental conditions in a continuously aerated, 5,000 L capacity fiberglass tank containing filtered tap water, feeding on a commercial diet, with a prior experimental feeding trial with powdered BSFL at four graded levels (0%, 10%, 20%, and 30%) for 21 days.

### Preparation of BSFL-supplemented experimental diet

The BSFL was procured from a local supplier. The samples were powdered, sieved, and placed in labeled, airtight plastic bags and stored at room temperature (26°C) until further use to make experimental pellets later. Different compositions of experimental feed containing specific materials were formulated using feed formulation software and weighed accordingly (Table-1) and mixed well based on isonitrogenous crude protein (30%) content (Table-1). In the formulation, six ingredients were in a fixed amount (fishmeal, fish oil, limestone, dicalcium phosphate, vitamins, and minerals), whereas three ingredients were variable (corn grain, soybean meal, and BSFL). The ingredients were combined with corn starch till a doughy consistency was achieved, and the mixture was pelleted using a manual pelleting machine. The pellets were dried in a 40°C oven for 2 days.

**Table-1:** Ingredients and composition (%, DM) of experimental diets formulation and BSFL.

Ingredients (g/kg)	BSFL level (%)			

	0	10	20	30

	Group A	Group B	Group C	Group D
Corn grain	15	16	13	10
Soybean meal	33	25	22	19
Fish meal	9.5	9.5	9.5	9.5
Fish oil	2.5	2.5	2.5	2.5
Limestone	2	2	2	2
Dicalcium phosphate	0.63	0.63	0.63	0.63
Vitamin	0.32	0.32	0.32	0.32
Mineral	0.32	0.32	0.32	0.32
BSFL meal	0	6.3	13	19

DM=Dry matter, BSFL=Black soldier fly larva

### Fish husbandry and feeding trial

The red hybrid tilapias were purchased from a private fish hatchery in Balakong, Selangor, Malaysia. They had a mean initial body weight of 1.46 ± 0.06 g and a mean initial length of 4.75 ± 0.56 cm. The fish were acclimated to a temperature of 30°C ± 1°C for 1 week before the experiment. After acclimatization, 120 fish were selected and allocated into four treatment groups, each with three replicates of the experimental feed formulation. The groups were labeled as Group A (the control group), Group B, Group C, and Group D. Each replicates (n = 10) was assigned to a tank measuring 2 m^3^ (1 × 2 × 1 m) and filled with 40 L of water. Each group of fish was fed twice a day with 5% of their body weight [14, 15]. Furthermore, the water was changed daily, and the tanks were cleaned once a week. Feed intake and fish growth were monitored bi-weekly by measuring assigned diets consumed and batch weighing fish per tank. Water quality parameters were maintained at optimal levels (dissolved oxygen and pH were maintained at 6.53 ± 0.41 mg L^1^ and 8.21 ± 0.32, respectively) in the culture tanks during the feeding trial. This was achieved by changing approximately 50% of the water once a week and simultaneously removing unconsumed diets and fecal matter at the bottom of the aquariums daily. Water parameters were checked on a daily routine and recorded (YSI 556: Handheld Multi-Probe Meter, USA).

The feeding trial was carried out for 21 days. The initial measurements of mean body length and mean body weight were taken at the beginning of the study on day 0, and ongoing measurements of length and weight were carried out on days 7, 14, and 21. At the end of the experiment, the number of fish per tank was counted and weighed individually to calculate survival and growth performance, respectively. All the fish sacrificed at the end of the study had their heads, fins, skin, and organs removed. The flesh was dried in an oven overnight at 60°C and then ground to a meal using a grinder.

### Proximate analysis of feed and fish meat

Proximate analysis of experimental feed was determined before the experiment, and fish carcasses were determined after the experiment using standard laboratory methods, according to the Association of Official Agricultural Chemists [16]. Dry matter (DM) and moisture content were determined after drying the sample overnight at 105°C until constant weight in a vacuum oven. Ash content was gravimetrically determined by incinerating the sample in a furnace for 4 h at 550°C in a muffle furnace. Crude fat content was determined using the Soxhlet fat analysis method. On a digital hot plate, crude fiber content was determined with successive hydrolysis with 0.26 N sulfuric acid (H_2_SO_4_) and 0.31 N sodium hydroxide (NaOH) for 30 min at 100°C. Crude protein content was determined using the Kjeldahl technique and multiplied by 6.25 factors [16].

### Calculations and statistical methods

To calculate various parameters, the following formulae were applied, where applicable;

1. Mean body length gained = Length final (Lf, cm) – Length initial (Li, cm)
2. Mean body weight gained (MWG) = weight final (Wf, g) – weight initial (Wi, g)
3. Specific growth rate (SGR, % per day) = [L_n_(Wf) − L_n_(Wi)/days]*100
4. FCR = feed distributed (g DM)/weight gained (WG)
5. Protein efficiency ratio (PER) = WG/total protein fed (g DM)

The data collected were tabulated in Microsoft Excel 2016 and expressed as mean ± standard error of the mean. Analyses were performed using IBM Statistical Package for the Social Sciences (version 25). One-way analysis of variance was performed for normally distributed data and the Kruskal-Wallis test for non-normally distributed data. Dunnett’s test was carried out to test the significance between the control and treatment groups at p < 0.05.

## Results

### Nutritional composition of experimental diet

Table-2 summarizes the results of the proximate analyses of the four experimental diets. According to the results, the feed formulation of Group D with the highest concentration of BSFL (30%) has the highest percentage of crude fat and protein content. Statistical analysis revealed significant (p < 0.05) differences in crude protein and fat content between the control and treatment groups.

**Table-2:** Nutritional composition(%, DW) of experimental feed.

Nutritional component (%)	Groups			

	A	B	C	D
Dry matter	99.35 ± 0.01^a^	99.44 ± 0.01^a^	99.42 ± 0.03^a^	99.52 ± 0.01^a^
Ash	14.06 ± 0.58^a^	16.01 ± 1.23^a^	12.81 ± 0.05^a^	13.01 ± 0.23^a^
Crude fat	2.33 ± 0.15^a^	6.26 ± 0.93^b^	7.99 ± 0.40^c^	12.60 ± 0.42^d^
Crude fiber	4.30 ± 0.01^a^	4.68 ± 0.15^a^	4.37 ± 0.43^a^	5.21 ± 0.17^a^
Crude protein	33.92 ± 0.31^a^	35.63 ± 0.66^b^	36.37 ± 0.01^c^	38.69 ± 0.35^d^

Different superscript lowercase letters on data per row indicate significant differences at p *<* 0.05, DW=Dry weight

### Growth performance

The results of mean body length, weight gained, SGR, FCR, and PER are tabulated in Table-3. The initial fish stocking density was 10 fish per aquarium tank. At the end of the experiment, there was no significant difference (p < 0.05) among all diets in terms of mean body length and weight gained. Every week, the weight gain of the fish was measured to determine growth. Fish-fed diets A, B, C, and D had initial mean weights of 1.38 ± 0.19 g, 1.51 ± 0.40 g, 1.42 ± 0.12 g, and 1.51 ± 0.11 g, respectively. At the end of the experiment, the mean body weight gain in Group D was the highest (1.53 ± 0.20 g), followed by Group C (1.25 ± 0.13 g), Group A (0.99 ± 0.33 g), and Group B (0.99 ± 0.33 g). The weight gain of fish was measured every week to determine growth.

**Table-3:** Body length and weight gained during the duration of the study.

Parameters	Group			

	A	B	C	D
Mean body length gained	0.80 ± 0.19^a^	0.36 ± 0.19^a^	0.93 ± 0.28^a^	1.10 ± 0.15^a^
Mean body weight gained	0.99 ± 0.33^a^	0.34 ± 0.05^a^	1.25 ± 0.13^a^	1.53 ± 0.20^a^
SGR (%/day)	1.97 ± 1.15^a^	0.76 ± 0.21^a^	2.20 ± 0.52^a^	2.70 ± 0.29^a^
FCR	1.47 ± 0.18^a^	1.67 ± 0.19	1.44 ± 0.56^a^	1.81 ± 1.38^a^
PER	0.23 ± 0.06^a^	0.21 ± 0.03^a^	0.32 ± 0.10^a^	0.25 ± 0.15^a^

Values are means ± SEM for triplicate experimental groups. Different superscript lowercase letters on data per row indicate significant differences, at p *<* 0.05, FCR=Feed conversion ratio, PER=Protein efficiency ratio, SGR=Specific growth rate

There were no significant differences (p < 0.05) in SGR, FCR, or PER across all diets. When compared to other diets, the fish in Group D had the highest SGR (2.70% ± 0.29%), and Group C recorded the best FCR (1.44 ± 0.56) and PER (0.32 ± 0.10).

### Nutritional composition of fish

Table-4 summarizes the results of proximate analyses of meat from each group. Group D had the highest crude protein content, at 79.21–81.39%, followed by Group C, which had a crude protein content of 71.29–76.89%. The crude protein content in Group A (control group) and Group B was 63.78–67.00% and 62.67–67.63%, respectively. Statistical analyses revealed significant differences (p < 0.05) in crude protein between Groups A, C, and D.

**Table-4:** Nutritional composition (%, DW) of fish fed with experimental diet.

Nutritional component (%)	Groups			

	A	B	C	D
Crude fat	0.71 ± 0.30^a^	0.67 ± 0.47^a^	2.15 ± 0.03^b^	2.90 ± 0.08^b^
Crude protein	65.39 ± 1.61^a^	65.15 ± 3.51^a^	74.09 ± 3.96^b^	80.30 ± 1.54^c^

Values are means ± SEM for triplicate experimental groups. Different superscript lowercase letters on data per row indicate significant differences, at p *<* 0.05, DW=Dry weight, SEM=Standard error of the mean

## Discussion

Growth performance in fish can be measured by increasing weight (WG% and SGR) or length, or by measuring both weight and length, in a relationship known as condition factor [2]. Optimal fish growth occurs when dietary requirements for protein, lipids, total energy, and a balance of vital amino and fatty acid, mineral, and vitamin concentrations were maintained. Moreover, growing awareness of the benefits of insect meal sparked an interest in using it in animal feed as they are a rich source of protein and fat, consume decaying organic matter, have a lower FCR, and emit fewer greenhouse gases [5]. The present study demonstrates that using 10–30% BSFL as a partial replacement for corn grain and soybean meal increased the crude protein and lipid content in the formulated diet. Further, the substrate on which the larvae are grown also influences the proximate composition of BSFL [17, 18]. The previous research discovered that larvae fed fruits and vegetables have lower crude protein and fat content than that fed manure [18, 19].

The growth performance was not significantly different between groups (p < 0.05). Group D (containing 30% BSFL) displayed the best growth performance followed by Group C (20% BSFL), and Group B had the lowest growth performance (10% BSFL). This could be because Groups C and D have higher protein and fat content than Groups A and B. These elements are critical in promoting the growth of red hybrid tilapia. Although statistical analyses revealed that the increase in growth was insignificant at p < 0.05, the results suggest that fish-fed BSFL diet performed slightly better in terms of growth. Inclusion of BSFL up to 30% in the commercial diet did not impair growth performance. Therefore, employing BSFL as an alternative protein source could potentially replace corn grain and soybean meal, thereby lowering feeding costs.

There were no significant differences in SGR across all treatments. These findings show that using up to 30% BSFL as a partial replacement for corn grain and soybean meal in the aquatic feed had no effect on fish – SGR. The best SGR value recorded was 2.70 ± 0.29% for fish in Group D. In terms of FCR value, there was no significant difference between all diets. However, when compared to the other groups, Group C (1.44 ± 0.56) produced the best value. A low FCR value is a good indicator of high-quality feed. This result suggests that BSFL could be used to feed red tilapia fingerlings as a 30% partial replacement for corn grain and soybean meal to achieve good feed utilization.

In this study, the nutritional composition of fish for crude protein and fat content revealed some significant differences between the control and treatment groups at p < 0.05. This finding contradicted previous research, which found that the diet composition did not affect protein and fat content in fish flesh [20, 21]. Nonetheless, the current determinations on how diet composition affected protein and meat content were consistent with other researchers’ findings [13, 22]. These discrepancies could be attributed to differences in the proximate composition of BSFL as a result of different rearing substrates.

## Conclusion

The study demonstrated that the inclusion of BSFL from 10% up to 30% in formulated diet did not impair the growth performance of fish. In addition, BSFL could be fed to red hybrid tilapia fingerlings as a 30% partial replacement for corn grain and soybean meal to improve feed utilization. The prospects for BSFL as a protein replacement for corn grain and soybean meal are promising, and additional research may help steer this sector toward long-term growth as the supply for seafood soars.

## Authors’ Contributions

HNA, HAH, and MFN: Designed the study, collected the data, interpreted the data, and drafted the manuscript. ZARA, WRH, SSA, and MYI: Designed the study, analyzed the data, and drafted and revised the manuscript. All authors have read and approved the final manuscript.
